# Supplementary material for: Are carbon emissions trading and green financial instruments synergistic? -Comprehensive quantitative research based on content analysis
Source: PLoS One. 2024 Mar 7;19(3):e0298601. doi: 10.1371/journal.pone.0298601 (PMC10919601; doi:10.1371/journal.pone.0298601)
Supplement: S1 Data — (ZIP) [file pone.0298601.s001.zip › Data/地级市Z_F工作报告.zip!/▒▒╛⌐/└┤╫╘╣1⁄2╓┌║┼í╛┬φ┐╦╩2╛▌═°í┐_2019.docx]

各位代表：

　　现在，我代表北京市人民政府，向大会报告政府工作，请予审议，并请市政协委员提出意见。

　　一、2018年工作回顾

　　2018年是贯彻党的十九大精神的开局之年，是改革开放40周年，是全面实施新一版北京城市总体规划的第一年。10月29日，习近平总书记主持中央政治局常委会会议，审议北京城市副中心控制性详细规划并发表重要讲话，充分体现了习近平总书记对首都发展、对北京工作的亲切关怀，为我们高质量规划建设管理好城市副中心指明了方向。

　　一年来，在以习近平同志为核心的党中央坚强领导下，在中共北京市委直接领导下，在市人大及其常委会监督支持下，我们坚持以习近平新时代中国特色社会主义思想为指导，深入贯彻党的十九大和十九届二中、三中全会精神，深入贯彻习近平总书记对北京重要讲话精神，坚持稳中求进工作总基调，贯彻新发展理念，落实高质量发展要求，全面深化改革开放，着力加强“四个中心”功能建设，提高“四个服务”水平，抓好“三件大事”，打好三大攻坚战，保持了经济社会平稳健康发展，较好地完成了市十五届人大一次会议确定的目标任务。初步统计，全市地区生产总值比上年增长6.6%左右，一般公共预算收入增长6.5%；居民消费价格上涨2.5%，城镇调查失业率控制在4.5%左右，全市居民人均可支配收入实际增长6.3%左右；万元地区生产总值能耗、水耗分别下降3.5%左右和3%以上，细颗粒物年均浓度下降到51微克/立方米。

　　(一)城市总体规划全面实施，疏解整治促提升专项行动成效显著。全面完成总体规划实施的45项年度任务，城市更新模式发生深刻变化，在城乡建设用地全年减量约34平方公里的同时，全员劳动生产率提高到24万元/人左右。建立市区规划分级管理体制，制定生态控制线和城市开发边界管理办法，完善“一绿”地区城市化实施机制，落实城乡建设用地增减挂钩。完善规划体系，发挥城市设计功能，编制完成分区规划，深化16个重点功能区综合提升方案和36项市级专项规划编制。开展天安门城楼及城台修缮，完成人民大会堂东广场改造修复和环境景观提升工程。发布实施历史文化名城保护发展规划，编制完成三条文化带保护建设规划，推进中轴线申遗保护。分别制定实施城市南部地区、新首钢地区、回龙观天通苑地区建设提升三年行动计划，城市发展的整体性协调性进一步增强。

　　坚持先立后破，广泛听取群众和专家意见，坚定有序推进疏解整治促提升专项行动，取得重要阶段性成效，市民满意率达九成以上。分类细化修订新增产业禁止和限制目录，退出一般制造业企业656家，疏解提升市场和物流中心204个。强化公共服务资源均衡配置，北京电影学院等新校区加快建设，天坛医院实现整体搬迁。整治“开墙打洞”8622处，中心城区29个老旧小区综合整治试点进展顺利。完成1141条背街小巷环境整治提升，核心区80公里道路电力架空线入地。拆违腾退土地6828公顷、还绿1683公顷，建成城市休闲公园28处、小微绿地121处，全市公园绿地500米服务半径覆盖率提高到80%。建设提升基本便民网点1529个，“一刻钟社区服务圈”城市社区覆盖率达到92%。

　　立足“一核两翼”联动发展，京津冀协同发展扎实推进。城市副中心行政办公区一期工程完工，按照“稳”和“俭”的要求，第一批市级机关正式入驻；城市绿心实现绿化1000亩，北京五中通州校区落成，友谊医院通州院区开诊。北京大兴国际机场正式命名，航站楼主体工程基本完工。全力支持雄安新区建设，4所对口帮扶学校挂牌，12家中关村企业入驻。制定实施推进协同发展新三年行动计划，与津冀分别签订新一轮战略合作协议，市域内国家高速公路“断头路”清零，联合推进永定河综合治理与生态修复，建立与密云水库上游地区的水源保护生态补偿机制，输出到津冀的技术合同成交额227.4亿元。

　　(二)营商环境明显改善，对外开放全方位扩大。全面贯彻落实习近平总书记关于北京要率先加大营商环境改革力度的要求，先后出台“9+N”系列政策措施和进一步优化营商环境三年行动计划。开办企业时间压缩至5天，成为全球开办企业完全免费的两个城市之一；率先开展小微企业获得电力“零上门、零审批、零投资”专项服务，每年为企业节省费用约12亿元；推进工程建设项目审批制度改革试点，搭建“多规合一”协同平台，社会投资项目许可办理时间缩减至45天；在国内22个城市营商环境试评价中综合排名第一，并为我国营商环境世行排名大幅提升作出北京贡献。

　　大力深化“放管服”改革，市区两级行政审批事项压缩一半以上，公布各级政府权力清单和行政审批中介服务事项清单、涉及企业群众办事创业证明目录，做到清单之外无事项、目录之外无证明。全面推进综合窗口服务模式，建成网上政务服务大厅，市区两级政务服务事项网上可办率均达到90%以上。项目竣工联合验收机制、涉税业务“全市通办”、市场监管“风险洞察平台”等5个典型经验在全国复制推广。

　　坚决贯彻落实习近平总书记在民营企业座谈会上的重要讲话精神，制定5方面69项政策措施，帮助民营经济解决发展中的困难。深入开展大走访大调研，为重点企业送上“服务包”，有针对性地解决企业用地、用房、人才引进等突出问题。建立纾困“资金池”，引导各类金融机构对接支持。

　　深入对接国际规则，服务业扩大开放综合试点三年两轮226项任务基本完成，累计形成68项开放创新成果，外资企业“全周期”管理、商务备案与工商登记“单一窗口、单一表格”受理等一批试点经验在全国推广。国内首家外资控股飞机维修企业落地运营，首家外资控股证券公司、三大信用评级机构等一批具有国际影响力的金融及服务机构落户北京，全年实际利用外资超过160亿美元。跨境电子商务综合试验区、深化服务贸易创新发展试点获批，服务贸易进出口额突破1万亿元。出台共建“一带一路”三年行动计划。积极营造适合国际人才的“类海外”环境，开展7家国际医院试点，办理港澳台居民居住证5895件，618名外籍高层次人才获得在华永久居留权。

　　(三)全国科技创新中心加快建设，高精尖产业发展态势良好。以国家战略需求引导创新方向，突破体制机制障碍，整合创新资源，打通创新链条，组建北京量子信息科学研究院、脑科学与类脑研究中心、智源人工智能研究院等新型研发机构，综合性国家科学中心建设全面展开。深化“三城一区”建设，高水平编制规划、细化方案。中关村科学城创新要素深度融合，服务创新主体能力增强，城市创新文化和形态更加优化，自主创新活力进一步提升，涌现出马约拉纳任意子、新型超低功耗晶体管等重大标志性原创成果。怀柔科学城综合极端条件实验、地球系统数值模拟等2个大科学装置和材料基因组等5个交叉研究平台建设取得阶段性进展；未来科学城着力引进开放性科研平台和双创平台；北京经济技术开发区利用外资水平加快提升，一批重大产业项目落地见效，创新型产业集群增势良好。设立规模为300亿元的科技创新母基金，专注布局硬科技和创新早期。全市国家高新技术企业达到2.5万家、增长25%，平均每天新设创新型企业199家，全市技术合同成交额增长10.5%。中关村示范区总收入超过5.8万亿元，独角兽企业80家、居全国首位。

　　深入实施新一代信息技术等10个高精尖产业发展指导意见，制定5G、人工智能、医药健康、智能网联汽车、无人机等产业发展行动计划和方案，精心谋划各区主导产业和重点培育产业方向。落实财政、土地、人才支持政策，推动土地弹性年期出让，实施中关村国际人才20条新政，2300多名人才办理引进落户手续。设立金融街服务局，着力提高国家金融管理中心服务能力。启动建设北京金融科技与专业服务创新示范区。制定防控金融风险三年行动计划，非法集资和互联网金融风险专项整治取得积极成效。规模以上高技术制造业增加值实现两位数增长，规模以上文化产业收入达到1万亿元，金融、科技、信息等优势服务业对经济增长贡献率达到60%以上。

　　(四)生态环境保护持续加强，城市治理能力进一步提升。落实全国生态环境保护大会精神，紧扣大气污染结构变化，坚持工程减排和管理减排并重，制定打赢蓝天保卫战三年行动计划，以钉钉子精神治理柴油货车、扬尘、挥发性有机物污染。坚持“周排名、月通报、季报告”，各级各部门环保责任进一步压实。淘汰国Ⅲ标准柴油货车4.7万辆，将低排放区扩展到全市域，排放超标检出率提高到15%。建成覆盖乡镇街道的粗颗粒物监测网络，道路清扫保洁新工艺作业率达到89%。利用热点网格技术精准执法，开展汽修、露天烧烤等专项执法检查，重点工业行业减排挥发性有机物2000吨。完成450个村“煤改清洁能源”，平原地区基本实现无煤化，清洁优质能源占比超过96%。修订实施重污染应急预案，抓早抓小，实现“削峰降速”。开展“清河行动”和水源地环境保护专项行动，完成非建成区84条段黑臭水体治理任务，全市污水处理率达到93%，重要水功能区水质达到国家考核要求，平原区地下水位回升近2米，密云水库蓄水总量超过25亿立方米。建成鲁家山餐厨垃圾处理厂等6处垃圾处理设施，垃圾分类示范片区覆盖率达到30%，生活垃圾资源化率达到58%。完成农用地土壤污染详查，基本建成土壤环境监测网络。实施新一轮百万亩造林绿化工程，新增造林26.9万亩。

　　坚持“控需、优供、强治”，大力开展交通综合治理，交通拥堵加剧趋势得到缓解。施行机动车停车条例，加大执法力度和频次，东城区、西城区和通州区支路以上路侧停车实现电子收费全覆盖。对全市345个堵点进行分级治理，完成年度治理任务，商务中心区、中关村软件园等一级堵点交通状况进一步好转。针对旅客打车难、候车环境差等问题，实施北京南站、北京西站、北京站71项整治任务，车站综合环境得到逐步改善。改善公交与地铁接驳换乘，优化调整公交线路93条，新增多样化线路89条，地面公交客运量止跌企稳。推动出台非机动车管理条例，开展违规销售电动车行为专项治理，下架违规电动车9500余辆。及时回应群众诉求，为174条无灯路段加装路灯，完成2700余处交通信号灯配时优化。6号线西延等3条轨道新线开通，轨道交通运营总里程达到636.8公里，共享单车监管服务平台投入运行，中心城绿色出行比例提高到73%。

　　积极推进“街乡吹哨、部门报到”改革，在16个区169个街乡进行试点，探索建立基层治理的应急机制、服务群众的响应机制、打通抓落实“最后一公里”的工作机制，得到中央深改委肯定。实施北京大数据行动计划，完成总体方案设计，推动市级机关政务数据上云应入尽入，在城市规划建设管理等领域开展试点应用。依托“12345”政府服务热线，有序推进各类政务服务热线整合。充分发挥《向前一步》公共政策对话节目作用，搭建市民参与城市治理的有效渠道。

　　(五)乡村振兴战略有力实施，精准扶贫脱贫持续发力。把乡村振兴战略摆在突出位置，实施美丽乡村建设三年专项行动计划。开展71个试点村村庄规划编制，带动全市乡村规划编制工作。抓好“四好农村路”示范区创建活动，升级改造农村公厕510座，完成1081个村庄人居环境整治任务。深刻汲取秦岭违建别墅问题教训，集中整治违建“大棚房”，持续推进浅山区违法占地违规建设专项治理。统筹推进承包地“三权分置”、闲置农宅盘活利用等农村各项改革，国有林场改革任务基本完成。创建北京农产品绿色优质安全示范区，平谷获批国家现代农业科技园区。出台推动生态涵养区生态保护和绿色发展的实施意见，组织开展平原区与山区、市属国企高校与低收入村结对帮扶，低收入农户收入增速持续快于全市居民。

　　认真落实中央东西部扶贫协作战略部署，聚焦精准扶贫脱贫，深入受援地精准对接，实施深化扶贫协作三年行动计划。加大资金项目、干部人才、产业就业、医疗教育等重点领域帮扶，投入59.5亿元，安排928个项目并全部开工。深入开展携手奔小康行动，构建企业和社会组织多方参与的大扶贫格局，全年助力受援地区60余万人脱贫。

　　(六)民生福祉不断改善，群众获得感进一步增强。切实办好老百姓家门口的事，老楼加装电梯等31件重要民生实事全部落实。坚持“房子是用来住的、不是用来炒的”定位，保持调控定力，商品住房价格保持稳定。全年分配公租房3.23万套(户)、网申共有产权住房2.9万套，其中30%的房源面向符合条件的非京籍家庭。新开工保障房5.45万套，完成棚户区改造3.43万户，均超额完成年度任务。完善城乡居民养老保障待遇确定和正常调整机制，新建养老服务驿站182家。建立居家养老服务津贴补贴制度，将老年人社会优待服务范围从65岁以上扩展到60岁以上。统筹整合困难群众救助政策，建立健全市区和街乡镇社会救助服务体系。完善促进就业创业政策，城镇新增就业42.3万人，农村劳动力转移就业6.4万人。首批6019人实现积分落户。

　　落实全国教育大会精神，深入推进教育体制机制改革。发布实施第三期学前教育行动计划，大幅增加学前教育投入，新增学前教育学位3万个。成立专职督查队伍，强化幼儿园监督监管。加强对中小学集团化办学和学区制管理的指导，开展小学和初中“三点半”课后服务，实施校外培训机构专项治理，支持和规范民办教育发展。统筹教育资源优化布局，在重点区域启动新建一批优质学校。深化医药分开综合改革，分级诊疗效果明显，建成34个紧密型医联体，一级医院及基层医疗卫生机构诊疗量增长近30%，三级医院门急诊量减少8%。全市95%以上的中小学校、托幼机构、养老机构食堂完成“阳光餐饮”工程建设。严厉打击非法“一日游”，投诉量同比下降67%，旅游秩序明显好转。加大文化惠民力度，实施营业性演出低票价补贴政策，151家实体书店获得房租补贴等专项支持。创建60个全民健身示范街道和体育特色乡镇，推动群众健身“六个身边”工程建设。举办市第十五届运动会和首届冬季运动会，成功申办2020年世界体育大会。

　　实施城市安全隐患治理三年行动计划，开展城乡结合部100个市级挂账重点地区综合整治，累计治理“三合一”场所和高风险群租房安全隐患2.6万余项，全市各类生产安全死亡事故起数下降18.5%。防汛减灾工作经受住多次强降雨的考验，实现了保安全、多蓄水。深入推进平安北京建设，开展扫黑除恶专项斗争，加强社会治安突出问题专项整治，深化矛盾纠纷排查化解，社会保持和谐稳定。支持国防和军队改革，配合驻京部队完成全面停止有偿服务任务，深入开展国防动员和双拥共建，军民融合发展成效显著。妇女儿童、残疾人事业取得新发展，民族、宗教、侨务工作进一步加强。

　　过去一年，我们认真履行首都职责，圆满完成全国“两会”服务保障工作，圆满完成2018年中非合作论坛北京峰会服务保障任务。基本完成2019年中国北京世界园艺博览会场馆和配套设施建设任务，“双100”招展目标全面实现，为如期开园打下坚实基础。

　　过去一年，我们扎实推进冬奥会、冬残奥会筹办工作，圆满完成平昌冬奥会、冬残奥会闭幕式接旗仪式和北京文艺表演，加快建设国家速滑馆、国家高山滑雪中心等新建场馆，启动国家游泳中心、国家体育馆等场馆改造，同步推进京张铁路、延崇高速等基础设施建设，兴延高速全线贯通。编制完成测试赛工作方案，全面推进市场开发。深入实施“共享冬奥”公众参与计划，成功举办第三届国际冬季运动博览会，冬奥宣传推广持续升温。

　　过去一年，我们认真学习宪法、遵守宪法，切实贯彻监察法，全面落实依法行政，制定修改废止政府规章49项，提请市人大常委会审议地方性法规6项，共办理市人大代表议案4项、建议931件，办理市政协提案941件。不折不扣落实中央批准的机构改革方案，基本完成市级机构改革。大兴调查研究之风，设立“无会周”，加大“四不两直”、蹲点调研力度，围绕重大部署、重点工作成立工作专班，以钉钉子精神狠抓落实。切实加强政府廉政建设，严格执行中央八项规定及其实施细则精神和市委贯彻落实办法，坚决防止“四风”反弹回潮，强化审计监督，努力营造风清气正的政治生态。

　　一年来，面对错综复杂的国际环境和艰巨繁重的改革发展稳定任务，取得这样的成绩来之不易。这是以习近平同志为核心的党中央坚强领导的结果，是习近平新时代中国特色社会主义思想科学指导的结果，是中共北京市委带领全市人民团结拼搏、努力奋斗的结果。在这里，我代表北京市人民政府，向全市人民，向全体人大代表、政协委员，向各民主党派、各人民团体和各界人士，向中央和国家机关各部门各单位、各兄弟省区市，向驻京解放军和武警部队官兵，向所有关心支持首都建设的香港特别行政区同胞、澳门特别行政区同胞、台湾同胞、海外侨胞和国际友人，表示诚挚感谢！

　　我们也要看到，当前我国发展仍处于并将长期处于重要战略机遇期，北京正处在城市更新、高质量发展的关键阶段，在面临新机遇的同时，也存在不少困难和挑战。主要是：人口资源环境矛盾等长期性、结构性问题依然存在；经济运行稳中有变，存在下行压力，财政收支矛盾凸显；原始创新能力不足和科技与产业创新脱节问题并存，科技创新支撑引领作用不够；供给侧结构性改革还不到位，服务业质量亟待提升，消费升级潜力没有充分释放；对外开放理念、能力和水平不足，与大国首都的地位和形象仍不相适应；治理“大城市病”任务依然艰巨，提高城市精治共治法治水平需要下更大功夫；发展不平衡不充分的一些突出问题尚未解决，公共服务供给还不能满足市民“五性”需求，城乡、区域协调发展水平有待进一步提升；政府系统形式主义、官僚主义现象依然存在，廉洁自律意识还需持续增强，工作能力和作风需要切实改进。对这些问题要高度重视，采取有效措施加以解决。

　　二、2019年主要任务

　　2019年是新中国成立70周年，是全面建成小康社会关键之年，大事多、喜事多，做好首都各项工作意义重大。我们要深刻认识和把握我国发展重要战略机遇的新内涵，积极主动应对新形势新挑战，化挑战为机遇，变外部压力为发展动力，坚定不移地抓住机遇、用好机遇、创造机遇，推动形成首都高质量发展的新优势。我们要切实承担起服务保障重大活动的政治责任，把新中国成立70周年庆祝活动作为全市工作的重中之重，统筹做好第二届“一带一路”国际合作高峰论坛等服务保障，高水平办好2019年北京世园会，全力筹办2022年冬奥会、冬残奥会，乘势而上，奋力拼搏，推动城乡面貌新变化、人民生活新改善，展现新时代首都新气象，向党和人民交上一份满意的答卷。我们要继续打好三大攻坚战，坚决防范化解金融、地方政府隐性债务等领域风险，守住不发生系统性风险的底线；坚决落实好对口支援、精准帮扶各项任务，助力受援地区贫困人口脱贫攻坚，抓好本市低收入农户脱低任务；坚决打赢打好蓝天保卫战、碧水攻坚战、净土持久战，不断提高城市精细化管理水平，为全面建成小康社会收官打下决定性基础，朝着国际一流的和谐宜居之都阔步前进。

　　今年政府工作的总体要求是：坚持以习近平新时代中国特色社会主义思想为指导，深入贯彻党的十九大和十九届二中、三中全会精神，深入贯彻习近平总书记对北京重要讲话精神，统筹推进“五位一体”总体布局，协调推进“四个全面”战略布局，坚持稳中求进工作总基调，坚持新发展理念，坚持推动高质量发展，坚持以供给侧结构性改革为主线，坚持深化市场化改革、扩大高水平开放，继续大力加强“四个中心”功能建设、提高“四个服务”水平，抓好“三件大事”，打好三大攻坚战，统筹做好稳增长、促改革、调结构、惠民生、防风险、保稳定各项工作，不断将全面从严治党引向深入，更加奋发有为地推动首都新发展，以优异成绩庆祝中华人民共和国成立70周年。

　　全市经济社会发展的主要预期目标是：地区生产总值增长6%-6.5%；一般公共预算收入增长4%；居民消费价格涨幅控制在3.5%以内；城镇调查失业率控制在5%以内；全市居民人均可支配收入增长与经济增长基本同步；万元地区生产总值能耗、二氧化碳排放分别下降2.5%和2.6%左右，万元地区生产总值水耗下降3%左右，生态环境进一步改善。

　　实现上述目标任务，重点做好以下工作。

　　(一)坚持规划引领，着力推动京津冀协同发展。

　　围绕落实城市总体规划，统筹推进疏解整治促提升专项行动，推动京津冀地区成为引领高质量发展的重要动力源。

　　抓好城市总体规划实施。严格审定落实分区规划，编制中心城区和新城控制性详细规划、专项规划和重点功能区规划，开展重点地区城市设计，建立总规引领、全域管控的空间规划体系。深入开展标准和政策规范制定工作，进一步完善规划管理机制，持续开展年度城市体检，为总体规划逐层逐级落实提供有力制度保障。制定城乡建设用地减量实施方案，全年减量30平方公里以上，推出一批城市更新试点区域和试点项目，推动城市减量提质发展。坚持“保障对保障”，按照申请式改善、“共生院”改造的思路，推进核心区平房院落有机更新。中心城区严格实行用地和建筑规模增减挂钩，对照规划要求抓好腾退空间使用。有序释放平原新城规划建设指标，提升规划建设标准和资源配置水平，提高综合承载能力。统筹生态涵养区的分类管控，严控开发强度，维护生态安全。

　　纵深推进疏解整治促提升专项行动。更加注重疏解整治与优化提升同步推进、疏解与环境整治紧密结合，加强政策与机制引导，进一步提升城市品质和改善人居环境。退出300家以上一般制造业企业，疏解提升市场和物流中心66家。调整优化公共服务资源空间布局，推动中心城区功能有序疏解，抓好同仁医院亦庄院区扩建、北京口腔医院迁建等项目，持续推进北京信息科技大学等5所高校新校区建设。持续深化违法建设治理，加大建筑垃圾资源化利用，按照“场清地净”标准拆违腾退土地4000公顷，引导鼓励各区争创无违建区、乡镇。基本实现违法群租房、地下空间散租、占道经营等动态清零，并建立长效机制。深入推进街巷治理，完成中心城区和通州区739条背街小巷环境整治提升任务。加大各区交界地区综合整治力度。按照街区生态补充社区菜店、早点、理发、维修、家政、快递等服务，鼓励便利店拓展服务内容，建设提升基本便民服务网点1000个。新建一批城市休闲公园、口袋公园、小微绿地，拆后还绿1600公顷。深入推进回龙观天通苑地区三年行动计划和三大攻坚工程，着力解决大型居住区群众关心的热点问题。

　　高质量规划建设管理好城市副中心。全面落实党中央、国务院批复精神，制定副中心建设指导意见、控制性详细规划实施方案和年度行动计划，创新副中心管理体制，提升综合承载能力，努力成为新时代城市建设发展的典范，成为新时代的精品城市。以市级机关搬迁为契机，推动适宜的功能和产业向副中心转移。启动行政办公区二期工程。全面实施城市绿心绿化工程，开工建设副中心站综合交通枢纽，高品质抓好运河商务区建设。推进环球主题公园建设，启动建设副中心剧院、图书馆、博物馆，抓好北京学校、安贞医院通州院区等一批公共服务项目建设，开展通州老城生态修复和城市修补，增强群众获得感。坚持“四个统一”，强化副中心与河北廊坊交界地区规划建设管控，推动交通基础设施、公共服务和产业向北三县延伸布局。

　　抓好协同发展重点任务落实。深入对接支持雄安新区，开工建设4所“交钥匙”学校医院，实现京雄城际北京段开通使用，力争京雄高速开工建设，切实做到雄安新区需要什么就主动支持什么。确保北京大兴国际机场如期建成通航，启动临空经济区起步区和综合保税区建设，实现外围交通市政配套同步投入使用。加快建设京沈客专、京唐城际等一批重大工程，推动试点大宗货物运输“公转铁”。深化三地产业政策衔接与园区共建，在区域内延伸创新链、产业链布局。进一步加强与河北交界地区生态环境管控，推进京津风沙源治理二期、京冀生态水源保护林建设。

　　全力做好冬奥会、冬残奥会筹办工作。扎实推进场馆建设改造，确保国家高山滑雪中心年底达到测试赛要求，做好2020年延庆高山滑雪世界杯等单项测试赛筹备，办好冰壶世界杯等国际赛事。建成京张铁路、延崇高速。加强冬奥人才引进和培养，组建示范场馆核心团队，有序推进赛事服务保障。着力提升城市无障碍环境。发布北京冬奥会、冬残奥会吉祥物。深入实施“科技冬奥”行动计划。持续推动冬奥知识、冰雪运动进校园、进社区，进一步营造期盼冬奥、参与冬奥的良好氛围。

　　(二)坚持改革开放，着力推动北京高质量发展。

　　认真贯彻“巩固、增强、提升、畅通”八字方针，深化供给侧结构性改革，落实好“六稳”要求，努力打造高质量发展的北京样板。

　　推进更高水平开放。对标国际先进理念、领先标准和最佳实践，全面落实新一轮服务业扩大开放综合试点方案，着力打造“负面清单+正向激励”“产业开放+园区开放”模式，加快教育、医疗等领域开放步伐。深化服务贸易创新发展试点，全面推进国际贸易“单一窗口”建设，完善协调管理机制，发挥综合保税区政策平台优势，提高口岸跨境贸易便利化水平。办好2019年京交会，扩大金融、科技、信息、文化创意、商务等领域服务出口，提高服务贸易国际竞争力。发挥国际交往中心资源优势，主动参与共建“一带一路”，建设对外交往、科技支撑、人文交流、服务支持4个重点平台，支持企业“走出去”。

　　进一步扩大金融业开放。继续加强金融街建设，服务好国家金融管理中心功能。在外汇管理改革、人民币跨境使用和投融资便利化等方面进一步推动符合首都金融业发展需求的措施落地，吸引国际知名银行、保险、证券、基金、资产管理等金融机构，以及会计、审计、评估、信用、法律、咨询等专业服务机构在京设立地区总部和分支机构。建立金融科技公共研发平台，金融科技与专业服务创新示范区建设要取得积极进展。积极推动“新三板”改革，提升融资能力和流动性，大力培育高精尖上市公司。严格履行地方金融监管和风险处置职责，深入推进互联网金融风险专项整治，持续严厉打击非法集资。

　　持续改善营商环境。深入落实优化营商环境三年行动计划，实施好北京效率、北京服务、北京标准、北京诚信四大示范工程。狠抓“9+N”政策2.0版落地，推动办理建筑许可、获得用水用气、纳税等领域明显改善。深化“证照分离”改革，切实解决困扰企业的准入不准营问题，扩大生活性服务业企业“一区一照”登记范围，持续优化企业注销登记。构建高效统一的税收征管体系，加快建设新一代电子税务局。深入开展政务服务“一网通办”，推动“网上可办”向“全程通办”深化，大力推进掌上办、自助办、马上办、就近办，让越来越多的事项实现“全市通办”。加快“一门”“一窗”办理，各级80%的事项实现“一门”办理，70%的事项“一窗”分类受理。更加注重用户体验，制定全市统一的政务服务事项规范和办事指南，在全市范围内努力实现同一事项同等条件无差别办理。进一步规范社区出具证明事项，企业群众办事提供的材料减少60%以上，各级100个高频事项实现一次不用跑或者最多跑一次。完善营商法治体系，推进社会信用立法，构建以信用为核心的市场监管体制。

　　坚定不移支持民营企业发展，出台促进民营经济健康发展意见，营造公平竞争市场环境。全面执行国家减税降费政策，确保企业社保缴费负担不增加。开展专项清欠行动，纠正一些政府部门和国有企业拖欠民营企业款项的行为。当好企业“服务管家”，确保兑现“服务包”承诺事项。充分发挥小微企业金融综合服务平台、企业上市综合服务平台、融资担保基金、纾困基金作用，降低小微企业融资成本。依法保护产权，进一步激发和保护企业家精神，着力构建亲清新型政商关系。深化国资国企改革，加快实现从管企业向管资本转变，抓好国有资本投资、运营公司试点。推动国有经济战略性重组，压缩企业管理层级，推进混合所有制改革，提升国有企业科技创新能力。

　　激发新一轮消费升级潜力。实施促进消费提升计划，出台“1+X”系列政策，进一步扩大商品消费和服务消费。开展生活性服务业品质提升行动，着力打造10条特色示范街区，培育100家连锁品牌企业。启动王府井等高品质步行街建设，实施传统商圈改造提升，推动商业企业转型升级。出台繁荣夜间经济促消费政策，鼓励重点街区及商场、超市、便利店适当延长营业时间。激发时尚消费、品牌消费，鼓励全球新品在京首发、开设首店，培育做优民族品牌、地方知名品牌。进一步升级信息消费，创建信息消费示范城市。抓好跨境电子商务综合试验区建设，新建10家以上示范体验店。继续实施节能减排促消费政策，扩充商品品类，发展绿色消费。破除各种隐性壁垒，完善土地规划、市政配套、执业环境、价格税费等政策配套，鼓励社会力量提供多层次多样化医疗、养老服务。支持发展国际化的专业和职业教育培训。扩大文化、体育、旅游消费，制定建设国际一流旅游城市意见，加强北京旅游境外宣传推广。推进全域旅游示范区创建工作，拓展精品旅游路线，在郊区试点布局建设精品酒店，提升京郊游品质和服务能力。积极推进新国展二三期建设，吸引国际国内知名会展在京举办，发挥优质会展对消费的拉动作用。落实好城市物流专项规划，推动物流配送仓储智能化、绿色化发展。建立健全相关标准规范，加强市场监管，提供高质量产品和服务。同时，发挥投资关键作用，优化投资结构，优先安排补短板、强弱项的投资，加强项目前期工作和储备，特别是做好吸引社会投资的工作。

　　(三)坚持创新驱动，着力增强全国科技创新中心的引领性和影响力。

　　以全球化视野谋划和推动全国科技创新中心建设，塑造更多先发优势的引领型发展，有力支撑首都高质量发展和创新型国家建设。

　　持续深化科技体制改革。充分发挥中关村示范区改革“试验田”作用，健全以知识价值为导向的分配制度，落实赋予科研机构和人员更大自主权政策，进一步深化职称制度改革。注重培养一批爱科技、懂创新、会服务的专业化机构和人才，建设创新网络服务平台，培育第三方服务市场，为创新型企业全生命周期做好优质服务。发挥科技创新母基金作用，进一步落实知识产权质押融资、投贷联动等创新举措，培育更多早投、长投科技型中小微企业的耐心资本，完善促进科技成果转化的政策、资金支持机制。加大政府采购首台套力度，为新技术新产品提供更多应用场景。加强知识产权保护和运用，完善知识产权公共服务体系，积极引进国际服务机构和法律人才。办好中关村论坛，拓展国际创新合作渠道，引进国际顶尖专业服务机构和孵化器，支持跨国公司在京设立研发中心，加快融入全球科技创新网络。实施“一带一路”科技创新北京行动计划，鼓励企业深度参与国际标准制定，不断增强北京在全球科技竞争中的影响力和话语权。

　　强化科技创新战略布局。建立基础研究长期稳定支持机制，用好北京自然科学基金，支持在京高校建设世界一流大学和一流学科，增强高校科技创新能力，提升前沿学科交叉和融合创新水平。积极承接国家重大科技任务，加快培育量子信息、网络空间安全等领域国家实验室，主动做好配套服务。深入对接国家科技创新2030-重大项目、重点研发计划和创新基地建设，争取智能制造和机器人、深空探测等重点任务和项目在京落地。在基础材料、光电子、高端芯片等重要领域，布局一批新型研发机构。聚焦5G、人工智能、医药健康等关键方向，精心组织推进创新攻关，更好服务国家创新战略需求。

　　努力打造国际人才高地。实施新时代推动首都高质量发展人才支撑行动计划，在全球范围内拓宽人才引进渠道，集聚培养一批战略科技人才、科技领军人才。加强和改进高校高精尖创新中心建设。推动中关村国家人才管理改革试验区建设，实施好雏鹰人才计划，重点支持一批30岁以下青年创新创业人才成长。加强青年企业家培训，办好企业家年会，着力搭建创新要素的融合与服务平台，促进企业家与投资人、科学家等深度合作。推进国际人才社区建设，打造国际人才港。

　　全力抓好“三城一区”主平台建设。在规划落实上下功夫，坚持产城融合，完善管理体制和市场化服务机制，推动科学城功能与科学、项目、产业等同步规划建设。中关村科学城要系统布局基础前沿和关键核心技术，构建城市创新文化和创新生态，努力培育世界一流科研院所，培育和发展具有全球影响力的创新型领军企业和“专精特新”的隐形冠军。怀柔科学城要深化城市设计，高质量落实控制性详细规划，建立完善高效、开放、可持续的管理体制和大科学装置运行机制，开工建设高能同步辐射光源等大科学装置以及第二批交叉研究平台，吸引研究机构、平台运营机构入驻并实体化运行。未来科学城要着力增强创新要素的活力，“一企一策”盘活入驻央企存量资源，鼓励和引导央企研发机构进行混合所有制改革，推动沙河大学城科教融合；特别是要全面加快中关村生命科学园三期建设，改革管理体制，重点抓好创新药研发。北京经济技术开发区要在管理体制、运行机制等方面实现突破，着力发展新一代信息技术、新能源智能汽车、医药健康、机器人和智能制造等四大产业集群，加快建设智能车联等20个技术创新中心和基因药物制备等10个产业中试基地。

　　不断壮大高精尖产业。狠抓10个高精尖产业发展政策落地，全面推行产业用地全生命周期管理，实施好差别化产业用地供地政策，积极盘活闲置低效用地和楼宇。加强先进制造业和现代服务业深度融合，统筹用好各类产业发展基金，促进制造业高质量发展。实施北京智源行动计划，推动人工智能带动各领域各产业升级和变革。推进医药健康协同创新行动计划，在研发外包服务、中试平台、研究型病房、创新品种审批绿色通道等方面取得积极进展。加快5G、工业互联网等新型基础设施建设，继续大力拓展各类创新技术的应用场景建设。推动奔驰新能源汽车、超高清显示设备、集成电路生产线、第三代半导体、“无人机小镇”等重大项目落地。搭建军民技术成果转化平台,打造军民融合特色园。编制中关村示范区一区十六园统筹发展规划，建立高精尖产业重点项目协调推进机制、跨区利益评估共享机制，引导各区、各园区聚焦主业、实现差异化发展。

　　(四)坚持问题导向，着力提升城市精细化管理水平。

　　深入贯彻落实习近平生态文明思想，提高“大城市病”治理的系统性和科学性，加快建设“天蓝、水清、土净、地绿”的美丽北京，不断提高城市治理水平。

　　持续抓好大气污染防治。坚持日常抓、抓日常，突出移动源、分散源污染治理，坚定不移打赢蓝天保卫战。把柴油货车治理作为重中之重，推动制定机动车和非道路移动机械排放污染防治条例，严格执法并实行闭环管理，鼓励淘汰高排放老旧柴油货车，支持发展新能源货车，实施非道路移动机械备案和环保标识管理政策。健全扬尘治理工作机制，在规模以上工地实现视频实时监控，严查严罚扬尘违法行为，全面加强裸地扬尘治理，实现各级各类道路清扫保洁全覆盖。深入推进挥发性有机物污染治理，对石化、印刷、汽修等重点行业开展专项执法检查。严防散煤复烧反弹，巩固无煤化治理成果。组建市区两级生态环境保护综合执法队伍，加大环境保护执法力度。认真落实空气重污染应急预案，加强区域大气污染联防联控联治。推广绿色生产和生活方式，鼓励群众监督举报。

　　加大交通综合治理力度。制定实施交通综合治理年度行动计划，努力改善绿色出行条件。在优化供给方面，统筹地面公交和轨道交通协同发展，完成全市公交干线网优化方案，落实分步实施计划，打造2条公交示范走廊；开通7号线东延、八通线南延、新机场线一期3条轨道新线，运营总里程达到699.3公里；完成长安街西延等主干路、25条次支路建设，打通一批“断头路”，推动代征代建道路移交；加强慢行系统网络建设，完成850公里自行车道治理。在强化治理方面，全市支路以上城市道路路侧停车位实现电子收费，加大违章停车执法巡查力度；加快智慧交通建设，更新完善一批科技设施，积极运用大数据、云计算等先进技术，整体提高交通治理科技水平；深化整治北京南站、北京西站、北京站交通转换环境，完善丰台火车站等新建火车站交通方案；落实学校、医院的主体责任，共同改善周边交通秩序；规范非机动车交通秩序，严格查处闯红灯、逆行等违规行为。在调控需求方面，规范网约车行业发展，鼓励共享出行，开展轻微型封闭货车综合治理。

　　进一步改善生态环境。注重提升森林质量，实施好新一轮百万亩造林绿化工程，全市新增造林绿化25万亩，推进温榆河公园、官厅水库八号桥湿地恢复等一批项目。坚持节水优先，珍惜用好南水北调水，完成5个节水型区创建。以“清河行动”和“清四乱”行动为抓手，严格落实河长制，加强重要河流综合治理和生态修复。严厉整治向城市雨水管道排污以及倾倒垃圾等非法行为。完成污水治理和再生水利用第二个三年行动方案，全市污水处理率超过94%。大力促进源头减量和垃圾分类，加快房山循环经济产业园等设施建设，生活垃圾分类示范片区覆盖率提高到60%，生活垃圾资源化率达到59%。编制农用地土壤环境质量分级清单，实施建设用地土壤风险管控。

　　提高城市精治共治法治水平。进一步深化“街乡吹哨、部门报到”改革，完善基层治理机制，落实网格化管理责任，更好发挥市民服务热线作用，提升服务群众的能力和水平。加快大数据管理平台建设，推动交通、政务服务、城市管理等领域大数据应用示范。建设城市管理专家智库，在城市街区更新、美丽乡村建设中落实责任规划师、设计师制度，提高街乡治理的专业化水平。及时回应市民诉求、媒体曝光和市民服务热线反映的问题，建立健全督查落实反馈机制，切实做到民有所呼、我有所应。调动各方积极性，发挥街巷长、小巷管家等作用，深化“门前三包”责任制，持续办好《向前一步》节目，推进共商共议共治。加强基层综合执法平台建设，推动执法力量下沉。对损害公共利益的违法行为，支持提起民事公益诉讼。

　　(五)坚持文化自信，着力抓好全国文化中心建设。

　　聚焦“一核一城三带两区”，提升城市文化品质，展现开放包容自信的大国首都人文形象。

　　加强社会主义核心价值观建设。紧扣重大纪念活动节点，开展主题教育、系列展览和文化活动。大力传承红色文化，推进香山革命纪念地保护利用。发挥首都志愿者带动作用，进一步推动学雷锋志愿服务制度化、常态化。开展向“北京榜样”学习活动，推动核心价值观融入日常生活。深化“五大文明创建”工作，助推石景山、顺义、怀柔、延庆创建全国文明城区，不断提升城市文明程度和市民文明素质。

　　推进历史文化名城保护。编制中轴线保护规划，扎实推进遗产点腾退、钟鼓楼周边疏解整治、正阳门到永定门步行环境提升等30余项重点项目。编制老城整体保护规划，开展文物腾退、历史文化街区划定等工作，保护好胡同肌理、四合院。完善文物保护工作机制，进一步明确文物使用单位责任，加强文物活化利用与安全管理。统筹推进三条文化带保护建设，加快推进大运河源头遗址公园、西周琉璃河考古遗址公园、长城保护等重点工程，提升“三山五园”地区整体环境。

　　提升文化服务质量。抓好文化惠民工程，支持博物馆、图书馆、文化馆、实体书店等文化设施建设，鼓励引导社会力量参与公共文化服务投入和运营，打造书香北京。加强对工业转型发展和疏解腾退空间的挖掘利用，扩展城市文化艺术空间。组织好传统节日活动，实施非遗传承人培育计划，推动优秀传统文化传承普及。抓好重大题材、现实题材和北京题材创作，推出一批体现新时代风貌、大国气派、首都特色的优秀文艺作品。

　　打造城市文化品牌。编制文化创意产业引领区建设发展规划，加快北京文化产权交易中心建设，推动“文化+”市场主体繁荣发展。支持老字号传承创新发展。制定文化特色小镇、文创街区建设导则，推动台湖演艺小镇、宋庄艺术创意小镇等重大项目加快建设。加强文化综合执法，净化网络空间和文化市场环境。办好北京国际电影节、国际音乐节、国际设计周和文博会等活动，提升城市文化开放性和国际影响力。

　　(六)坚持城乡区域协调发展，着力推动乡村全面振兴。

　　深入把握“大城市小农业”“大京郊小城区”的市情和乡村发展规律，走具有首都特点的乡村振兴之路。

　　深入落实乡村振兴战略。坚持因地制宜，体现京韵农味，突出多样性、特色化，加强对村庄风貌、建筑风格和色彩的管控引导，年内完成1000个左右村的规划编制工作。实施“百村示范、千村整治”工程，下大气力抓好农村垃圾污水处理、“厕所革命”、村容村貌整治、“四好农村路”等任务，不断改善农村人居环境。继续推动农村土地制度三项改革试点，基本完成全市农村集体资产清产核资。深化国家农业科技园区和农产品绿色优质安全示范区创建和升级工作，推动都市型农业高质量发展，促进一二三产业融合发展和农民增收。完善支持人才到农村创新创业的政策措施，鼓励城市各类人才带着资金、技术下乡创业。推进基层干部人才培养工程，培育科技型农民、管理型农民和农村实用技术人才，为乡村振兴提供人才支撑。坚决杜绝违建“大棚房”问题反弹，继续整治浅山区违法占地违规建设。抓好非洲猪瘟有效防控，确保疫情不扩散不蔓延。推进新一轮山区农民搬迁工程，启动实施2400户、5300人的搬迁任务。落实低收入农户精准帮扶措施，进一步提高教育、医疗、住房“三保障”水平，基本完成农村四类重点群体危房改造任务。

　　积极推动重点区域建设。实施新一轮城市南部地区加快发展行动计划，加快建设南水北调大兴支线等市政设施，实施南苑森林湿地公园等生态环境项目，提升大兴、亦庄、房山等南部新城的综合承载力。以长安街西延线、永定河两岸为重点加强城市设计，基本完成首钢南区规划优化，加快推进冬奥广场片区改造建设，努力打造城市更新标杆工程。推进“一绿”地区城市化建设，探索“二绿”地区减量发展新路径。落实生态涵养区生态保护和绿色发展的实施意见，完善平原区与生态涵养区结对协作机制，鼓励延庆、怀柔、密云等区加快创建国家森林城市，通过设立绿色发展基金、发行绿色债券等方式吸引社会资本加大投入，推动生态涵养区成为绿色发展的示范区。

　　扎实做好扶贫协作和支援合作。坚持精准扶贫脱贫方略，认真落实三年行动实施意见和行动计划，深化细化资金项目、干部人才、产业就业、医疗教育等帮扶工作。推动结对帮扶向行政村、学校、医院等基层单位延伸。加强受援地区干部人才培训，培养农民合作组织和致富带头人。建立防止返贫机制，再助力一批受援贫困县脱贫摘帽。深化南水北调对口协作，继续做好对口合作和区域合作。

　　(七)坚持以人民为中心，着力推进民生保障精准化精细化。

　　紧扣“七有”要求和“五性”特点，完善制度、守住底线，扎实办好重要民生实事，让群众有更多、更直接、更实在的获得感、幸福感、安全感。

　　优先发展教育事业。落实《首都教育现代化2035》及五年实施方案，把立德树人根本任务落到实处。继续实施第三期学前教育行动计划，加强普惠性民办园教师工资指导，强化幼儿园监管，新增学位3万个。平稳推进考试招生制度改革，加强教育督导。强化优质基础教育资源布局的市级统筹，加大对新城、重点区域和农村地区中小学校支持力度。拓展中小学教师来源，推进绩效工资改革。继续实施校外培训机构专项治理。推动市属高校内涵、特色和差异化发展，完善沙河、良乡大学城的科学规划和空间规划。促进具有首都特色的现代职业教育和民办教育健康发展，办好民族教育、特殊教育、网络教育和继续教育，建设学习型城市。

　　积极做好就业和社会保障工作。把稳就业放在更加突出位置，加强就业形势监测和公益性岗位应急储备，支持企业稳定岗位，抓好重点群体精准就业帮扶。加强市场价格监测预警，做好物价调控工作。落实企业职工基本养老保险基金中央调剂制度，鼓励企业年金、个人储蓄性商业养老保险发展。抓好社会救助综合改革试点，推进低收入农户帮扶措施与社会救助政策全面融合。推进医养结合，健全“三边四级”养老服务体系，实施差异化养老机构运营补贴政策，新建150家养老服务驿站。维护妇女儿童、未成年人合法权益，促进残疾人社会融合。

　　更好解决群众住房问题。完善住房市场体系和住房保障体系，保持房地产市场平稳健康发展。完成1200公顷住宅供地，加快已供地住宅项目开工建设和入市步伐。出台进一步规范管理住房租赁市场政策措施，促进住房租赁市场稳定。加强老旧小区改造工作统筹，多渠道建设筹集租赁住房5万套(间)、政策性产权住房6万套，完成棚户区改造1.15万户。

　　持续推进健康北京建设。推进医耗联动综合改革，抓好医疗服务价格动态调整和公立医院薪酬制度改革试点。加强预约挂号管理，扩大专家团队服务模式，严厉打击“号贩子”和“网络医托”，查处骗保行为，持续改善就医秩序和就医环境。继续推进分级诊疗，建设100个国家级临床重点专科医联体服务点，提升院前急救水平。强化全科医生培养与使用激励，推广“智慧家医”服务，提高基层医疗服务能力和水平。加强慢性病综合防控示范区建设，推进中医药服务“身边工程”，提升预防、治疗、康复综合服务水平。强化食品药品全过程监管，确保16个区全部达到国家食品安全示范城市创建标准。铺设100公里健走步道，建设维护好群众身边的体育设施。办好2019年男子篮球世界杯。

　　全力维护社会和谐稳定。深化街道管理体制改革，开展新一轮社区减负增效，完善社区治理，建设和谐社区。完善首都应急管理体系，加强应急指挥系统和应急救援队伍建设，提高防灾减灾救灾能力。有序推进城市安全隐患治理，建立隐患台账，完成年度消隐任务，严防重特大事故发生。切实加强平安校园建设。坚持和发展“枫桥经验”，发挥信访工作联席会议机制作用，深化矛盾纠纷排查调处，加大信访积案化解力度。全面贯彻党的民族、宗教政策，深入开展民族团结进步创建活动，促进宗教关系积极健康。严格落实反恐防恐“六住”措施，纵深推进扫黑除恶专项斗争，依法严厉打击各类违法犯罪活动。

　　全力支持国防和军队改革发展建设，深化国防动员和后备力量建设，做好退役军人服务管理工作，维护军人军属合法权益，不断巩固首都军政军民团结的良好局面。

　　三、加强政府自身建设

　　进一步增强责任感和使命感，自觉践行以人民为中心的发展思想，切实转变职能，狠抓工作落实，创造性地开展工作，建设人民满意的服务型政府。

　　坚持正确政治方向。树牢“四个意识”，坚定“四个自信”，坚决维护习近平总书记党中央的核心、全党的核心地位，坚决维护党中央权威和集中统一领导，切实做到“三个一”“四个决不允许”。不折不扣落实“看北京首先要从政治上看”的要求，更加主动地把北京发展放在党和国家发展大局中去定位、去谋划，始终从政治上把大局、看问题、做工作。按照中央统一部署，精心组织开展“不忘初心、牢记使命”主题教育，教育引导党员干部悟初心、守初心、践初心。

　　全面推进依法行政。认真执行市人大及其常委会的决议和决定，落实重大决策出台前向市人大常委会报告制度，自觉接受人大和政协监督。支持监察机关依法独立行使监察权，主动接受监察监督。紧扣城市转型发展需要，推动城乡规划、科技成果转化、公共数据共享开放等重点领域立法。健全依法决策机制，发挥法律顾问制度作用，做好决策合法性审查工作。加强执法规范化建设，推进行政执法公示、执法全过程记录和重大执法决定法制审核等制度，健全执法过程考核机制，推进行政执法与刑事司法有机衔接。加强行政复议和应诉工作。加大法律培训力度，提升基层依法行政能力。落实普法责任制，推进公共法律服务体系建设。

　　坚决落实机构改革任务。以推进机构职能优化协同高效为着力点，深化转职能、转方式、转作风。抓好市级部门“三定”规定的落实，调整优化部门所属事业单位。明确机构改革后各部门权力清单、责任清单，加强部门综合窗口能力建设，提高服务质量和效率。深化行政执法体制改革和事业单位分类改革。规范各类派出机构设置，组织实施好区级机构改革。

　　持续改进工作作风。强化质量意识、效率意识、成本意识，把高质量抓落实作为改进工作的重要方向。树立过紧日子的思想，一般性支出压缩幅度不低于5%。全面实施预算绩效管理，在重点公共服务领域开展绩效成本预算试点，提高财政资金使用效率。加强全口径政府债务管理，严控新增隐性债务、稳步消化存量。继续实行“无会周”，深入基层蹲点调研，增强调研的针对性和实效性。坚决破除形式主义、官僚主义，大力精简各类检查、报表、会议，让基层干部有更多时间和群众在一起，发现问题、解决问题。健全“督考合一”，把专业督查和第三方评估相结合，提高群众满意度在绩效考评中的占比。完成北京市第四次全国经济普查。贯彻执行新修订的公务员法，关心关爱干部，进一步激励广大干部新担当新作为。

　　着力加强廉政建设。学习贯彻《中国共产党纪律处分条例》，持之以恒落实中央八项规定精神，全面履行从严治党主体责任，坚定不移把政府系统党风廉政建设和反腐败工作推向深入。聚焦突出问题、紧盯关键节点，下大气力解决“四风”问题。深入开展廉政教育和廉政文化建设，防止“为官不为”“为官乱为”，严防发生侵害群众利益的不正之风、腐败行为和“小官贪腐”等问题。建立政务公开地方性标准，全面推进阳光政务建设。拓展审计监督的广度和深度，推动公共资金、公共资产、公共资源和领导干部履行经济责任情况审计全覆盖，促进权力规范运行。

　　各位代表！新时代是奋斗者的时代。让我们更加紧密团结在以习近平同志为核心的党中央周围，高举中国特色社会主义伟大旗帜，在中共北京市委的坚强领导下，以锐意进取、永不懈怠的精神状态，以改革创新、勇往直前的奋斗姿态，凝心聚力，真抓实干，奋力谱写中华民族伟大复兴中国梦的北京篇章，以优异成绩迎接新中国成立70周年！
